# Understanding the Importance of Gene and Environment in the Etiology and Prevention of Type 2 Diabetes Mellitus in High-Risk Populations

**Published:** 2016-03-14

**Authors:** Marsha Samsom, Tushar Trivedi, Olubunmi Orekoya, Shraddha Vyas

**Affiliations:** 1 Arnold School of Public Health, University of South Carolina, Columbia, SC, USA; 2 Cancer Prevention and Control Program, Arnold School of Public Health, University of South Carolina, Columbia, South Carolina, USA

**Keywords:** Diabetes mellitus, High-Risk, Genetics, Screening

## Abstract

Current literature focuses on the complications and treatment of Type 2 Diabetes Mellitus (T2DM) while clustering environmental and genetic factors to explain the disease. Interventions proposed to reduce diabetes prevalence should focus predominantly on initiating active rapports of family members and promoting a more communication-oriented preventative approach between diabetics and non-diabetics. Due to varying risks in T2DM by race and ethnicity, these populations should follow race-appropriate guidelines to prevent further T2DM occurrence and complications. The review consists of information related to the genetic component of T2DM to help identify high-risk groups and focuses on the environmental aspect of the disease to help consider appropriate techniques to reduce disease burden. Genetic factors play important roles in the pathogenesis of diabetes and thus are an essential element of understanding the cause of the disease and possible methods of prevention. Focusing on high-risk groups with T2DM could have profound effects on the current health care situation. In this review, we discuss the epidemiology of diabetes in the United States and propose methods of preventing and delaying the development of T2DM in high-risk individuals.

## Introduction: Disease Etiology

Type 2 diabetes mellitus (T2DM; previously known as non-insulin dependent diabetes mellitus) is a major public health threat affecting approximately 25.8 million people in the U.S. of all ages. It accounts for more than 90% of all diabetes and has a prevalence of approximately 8% among U.S. adults [1]. Prevalence of T2DM appears to be increasing in the U.S. and one of the major risk factors contributing to this increase is obesity in the population [2]. T2DM is a metabolic disease with varying degrees of insulin resistance and impaired insulin secretion from pancreatic islet beta cells, which causes hyperglycemia or high blood glucose [3]. Common early presenting symptoms of T2DM include polyuria (frequent urination) and polydipsia (increased thirst).

Pre-diabetes is a precursor to T2DM; it is a milder condition where individuals have higher blood glucose levels than recommended but do not meet the criteria for T2DM [4]. These individuals are at higher risk of developing diabetes, as well as other chronic diseases like stroke and heart disease. However, proper dietary management and increased physical activity can reverse pre-diabetic conditions [5,6]. Other known modifiable risk factors for developing pre-diabetes or T2DM are obesity, poor diet, and sedentary lifestyle [7].

The burden of T2DM varies drastically by race. In 2007–2009, the national survey data reported approximately 7.1% non-Hispanic white, 8.4% Asian Americans, 11.8% Hispanic/Latinos, and 12.6% non- Hispanic blacks had diabetes [8]. Although there have been continuous public health efforts to reduce T2DM rates, the prevalence of T2DM has been rising [9]. Studies have shown that genetic components and environmental factors play an important role in the pathogenesis of T2DM. To address the increasing rates of T2DM, it is important to understand this heterogeneous condition and to suggest practical methods of prevention by race and/or ethnicity. The prevalence of diabetes is much greater in minority populations who may necessitate novel and direct methods of prevention.

In 2005–2008, a study showed that the percentage of U.S. adults 20 years of age and older with pre-diabetes were similar for non-Hispanic whites, non-Hispanic blacks, and Mexican Americans. 8 Despite these similarities, T2DM rates are much lower for whites than minorities. A possible explanation is a lack of information about high-risk groups to prevent the onset of T2DM (i.e. differences in population screening). Currently, the U.S. preventive services task force (USPSTF) recommends screening for T2DM in asymptomatic adults with sustained blood pressure greater than 135/80 mm Hg [10]. More strict guidelines are needed to increase T2DM prevention. Interventions proposed to reduce diabetes prevalence are initiated through active engagement of diabetic populations, their family members, and the overall community. However, this strategy is currently neglected in high-risk groups, which reduces its potential for reducing incidence and mortality. The current literature does not adequately assess the importance of screening for high-risk groups or the success of these interventions in high-risk groups specifically.

Diabetes is the seventh leading cause of death in the U.S. Uncontrolled T2DM can lead to severe health consequences like kidney failure, amputations, and blindness; In addition to the individual complications of this illness, T2DM causes a significant economic burden with the total estimated cost of diagnosed T2DM costing $245 billion in 2012 [11]. The effects of T2DM require us to examine the literature in order to understand which methods will have the most positive effects in reducing the financial and social burden of T2DM. The purpose of this article is to 1) review the current literature surrounding the genetic and environmental components associated with T2DM and to 2) highlight the need for specific preventive measures in high-risk groups.

## Natural Trajectory of T2DM

The natural trajectory of T2DM may differ between individuals based on family history and environment. It is important to differentiate individuals depending on their risk of disease to determine possible screening and prevention methods that may lead to a reduction in the prevalence of T2DM in the United States.

The typical form of T2DM is divided into multiple clinical phases. In the beginning, hormone effects do not alter the target tissues for insulin action but as the disease progresses there is an increase in insulin concentrations and postprandial hyperglycemia occurs. This is followed by fasting hyperglycemia, and long-term complications of atherosclerosis, peripheral neuropathy, renal disease, cataracts, retinopathy and overt diabetes. Complications are due to genetic predisposition and metabolic control and can be controlled with monitored blood glucose levels [12]. Major phenotypic features of T2DM include age at onset, hyperglycemia, relative insulin deficiency, insulin resistance, obesity and acanthosis nigricans (skin disorder) [13]. Except under conditions of physical duress, type 2 diabetics rarely develop ketosis.

Although typically associated with increasing age, T2DM incidence is increasing among children and teenagers [14]. The proportion of individuals with the presence of a particular disease-causing genotype that has any symptoms of the disease is referred to as penetrance [12]. The high rate of genetic penetrance and more severe forms of T2DM is usually associated with obesity and sedentary lifestyle [15,16]. With lifestyle modifications, T2DM can be prevented or controlled. T2DM may have varying expressivity, which may cause an individual to have more symptoms or complications than another [12].

Researchers recently identified an unusual subtype of T2DM: maturity-onset diabetes of the young (MODY). Unlike the general form of T2DM, MODY is clinically present in adolescents and young adults who are not obese with a penetrance exceeding 80% [15,17] and follows an autosomal dominant inheritance. The MODY transcription factor is usually encoded on chromosome [12,15] and MODY is associated with mutations from at least nine genetic loci. With the increasing prevalence of T2DM and newly emerging forms, it is critical that scientists propagate information to increase awareness and reduce disease prevalence. Targeting these groups in addition to traditional high-risk groups could have significant implications for reducing T2DM prevalence.

## Role of Genetic and Environmental Factors

### Genetic role

Genetic factors play important roles in the pathogenesis of diabetes. Over the last two decades, many of the Mendelian disorders that presented with diabetes as one of their phenotypic features have been identified and characterized at a molecular level. Previous studies have identified mutations in specific genes that were associated with disorders of glucose homeostasis with a Mendelian pattern. Few examples include PPARG mutation in familial partial lipodystrophy (FPLD3)[18], INSR in Donahue syndrome and Type A insulin resistance, HNF4A in Maturity-Onset Diabetes of Young (MODY1) [19,20], and INS in Diabetes-type hyperglycemia and hyperinsulinemia [19,21].

Although certain common single nucleotide polymorphism (SNP) appear to increase the risk of T2DM [22], genetic variants that underlie increased susceptibility to T2DM at the population level are not clear. This is partly because T2DM is a heterogeneous condition that may involve several molecular pathways. The evidence that genetic factors play important roles in the pathogenesis of T2DM is illustrated when examining familial aggregation and the prevalence across populations. White and Asian populations have a low prevalence compared to Hispanics and African-Americans [12,23]. Furthermore, monozygotic (MZ) twins show higher rates of concordance than dizygotic twins.^3^ The odds of disease prevalence in families are about 4 times greater than the odds of the overall population, and the heritability of T2DM can be as much as 80%. Despite strong genetic influences, T2DM disease concordance is less than 100% in MZ twins and strong evidence that non-genetic factors (environmental) play a role in the illness [12]. A recent study in Finland concluded that the highest heritability based on all patients with T2DM and their family members was found in age groups 35–60 with 0.69 ± 0.16 heritability [19].

### Environmental role

Individuals are exposed to three major types of environment: the physical environment, the “built” environment, and the food environment. Physical environment exposures can include deleterious chemicals such as estrogen mimetic and bisphenol A [BPA], which are associated with hormone regulation, development of obesity and diabetes.

The “built” environment refers to the patterns of human behavior (urban design, land use, and transportation systems). As seen in the study by Ershow, a 10% increase in land use (density of commercial structures) can lead to an increased density of fast-food outlets, obesity, and T2DM [24]. Regulators of the food environment assume that, with guidance for lifestyle modification, individuals will make the proper decisions for their health to manage their disease. However, the nutritional information available often does not meet the minimum requirements for comprehension by the mass public. The Nutrition Facts Panel data requires literacy and numeracy skills to range from the 6–9th grade. Yet, most adult Americans fall short of this. For obese individuals and people with diabetes, making a lifestyle modification choice will not be readily attainable if they lack the educational prowess, which can inhibit self-confidence and reduce the odds of success [24]. Furthermore, the physical, built, and food environment can lead to decreased hours of sleep, which is of public health concern. Decreased sleep is associated with developing obesity and diabetes [24].

These studies show the importance of incorporating the genetic and environmental component of diabetes. Neglecting related factors may have deleterious effects on research progress and health outcomes.

## Identification and Screening of High-Risk Groups

### Identifying high-risk groups

Using a cohort of 15,000 patients from 2003–2007, the University of Wisconsin Health innovation program showed that more than 40 percent of minority patients who were at high risk for diabetes were not screened [25]. These patients had access to insurance and were categorized based on several American Diabetes Association (ADA) risk factors, such as age, high blood pressure, cholesterol levels, history of pre-diabetes, obesity, heart disease and race/ethnicity. To determine individuals at high-risk, preliminary questionnaires, such as the noninvasive Finnish Diabetes Association Type 2 Diabetes Risk Assessment Form questionnaire (FDATRA) [26] and the International Diabetes Federation (IDF) questionnaire, can be utilized. The latter identifies those who may be at risk based on age, ethnicity, waist circumference, family history, cardiovascular disease, gestational and drug history [27].

The reliability of screening for undiagnosed T2DM using the FDATRA form questionnaire has been validated in previous studies of certain minorities like the Phillipinos [26]. Future studies should consider validating these forms for high-risk groups in the U.S., such as Hispanics and African American groups.

The population at risk varies by geographic location and the population make-up. Differences in access to quality care, social and cultural factors, or the genetic make-up of distinct populations in an area can affect population risk. Between 1998 and 2002, the prevalence of T2DM among Hispanics varied from 6.2% in Illinois to 9.3% in Puerto Rico [28]. An American male’s lifetime risk is 1 in 3 and for a female it is 2 in 5 [29]. Further, in minorities like Hispanic women, the lifetime risk of developing T2DM is as high as 1 in 2.30 The lifetime risk of developing T2DM is about 40% if one parent has the disease (greater if the mother is affected) and close to 70% if both parents have diabetes [30].

Previous research has shown that the most efficient way of preventing T2DM is by intervening directly. In a study by Nishigaki et al., parents with diabetes were surveyed about the importance of communicating with their offspring about T2DM prevention. Eighty-three percent of patients thought it was necessary to advise their offspring, but only 62% gave advice to their child about diabetes prevention, management, and treatment The odds of giving advice to offspring increased when the patients were females, living with the offspring, had the disease for a longer duration, had taken insulin treatment, experienced complications and/or received oral medications. Patients communicated with their children about T2DM if they had detrimental health status, perceived their risk as being possibly debilitating, and had a personal relationship with their child [31].

## Screening High-Risk Groups

Individuals are currently screened for T2DM based on risk factors, such as age, sex, obesity, low physical activity, smoking, diet, and ethnicity; history of gestational diabetes mellitus, elevated blood pressure, family history, and elevated levels of non-diabetic fasting. Screening in asymptomatic populations is based on the prevalence of these risk factors [32]. The best screening test for T2DM is the fasting plasma glucose test (FPG test). It is inexpensive, quick and often performed for diagnostic testing, too. Pre-diabetes is labeled as an FPG of 100 to 125 mg/dL and diabetes is 126 mg/dL or above.^33^ Another test often performed is the oral glucose tolerance test (OGTT) with pre-diabetes ranging from 140–199 mg/dL and diabetes 200 mg/dL or above [33]. This test is usually performed if the FPG test is small, but the individual has a high risk of being diabetic. The third test, Hemoglobin A1C (A1C), is a valuable tool to diagnose, previously only used for educational purposes. An A1C ranging from 5.7–6.4% signifies pre-diabetes and 6.5% and above demonstrates diabetes [32,34]. The higher the value of the FPG, OGTT, or A1C test the greater the risk of the individual to develop diabetes or present with diabetes.

Other methods that have been utilized in research include testing for certain genetic variants. Many SNPs are associated with an increased risk of T2DM, but screenings for these variants have only been minimally beneficial in predicting the disease at the population level. Combining these SNPs with clinical characteristics may be an effective method of screening high-risk individuals [3]. Identifying genes that play a role in diabetes expression can increase our overall understanding of specific biological pathways and further develop targeted preventive measures. Scanning the genome for susceptible T2DM mutations and SNPs were performed for more than 20 different populations and suggested that the susceptible loci reside on multiple chromosomes. The region associated with T2DM varied by population. The most consistent findings, which are likely to contribute to the nature of the T2DM pathogenesis, were found on chromosome 1 (region 1q21-q25) [3]. Much is unknown about the exact role of this chromosome, but the primary report of this link was found through the Pima Indians. Since then there has been enough evidence, in many cultures, of its role in T2DM [3]. Important tools that have enabled further findings in the field include oligonucleotide and cDNA microarrays. These tools have permitted the study of discrepancies in gene expression of cells and tissues.

Researchers warn of treating separate disease loci as independent of each other because the complex interactions of genes and environment might be masked. These interactions may be crucial to understanding the final expression of the disease. For example, statistical analysis shows a significant interaction between chromosome 2 and 15 in determining T2DM susceptibility but conventional analysis continuously fails to analyze these chromosomes together [3].

In addition to considering single candidate genes, it is important to understand the interactions between genes. Scientists have identified many genes as being a significant contributor to T2DM by race/ethnicity, including Mexican American: chromosome 2q, 3p, and 10q; French Caucasians: chromosome 3q; Chinese: chromosome 6q; American Caucasians: chromosome 8p and 20q; and Finnish Caucasians: chromosome 12q and 18p. A weaker link has been identified among different populations and these chromosome locations [3].

Certain populations have more associated chromosomes related to T2DM, such as the Hispanic population [3]. There are several susceptible genes associated with T2DM such as PPARγ located on 3p25, which increases the risk of T2DM by up to 3 folds. Other gene locus variants on the 11p15 [1]. Are ABCC [8] and KCNJ [11], have shown to increase the risk up to 4 times [35]. These were identified based on the genes interaction with the pancreatic β cell function. Furthermore, differences in T2DM susceptibility exist across various ethnicities, environments, and gene-environment interaction.

## Conclusion

Currently, the primary modes of prevention include screening for disease and attacking the common risk factors for the disease [36]. Possible medications to treat or manage T2DM include oral hypoglycemic agents, such as sulfonylureas; a third class of agent that binds to PPARG to reduce insulin resistance; and a fourth class that acts on the intestinal absorption of glucose. These classes can be used independently or in conjunction depending on the severity of the disease [12]. However, it is estimated that only 16% of type 2 diabetics in the US take medications to control their disease. These drugs can be more effective by adhering to healthy lifestyle behaviors such as physical activity, proper nutrition and/or weight loss and may even become unnecessary in some cases [12].

High-risk groups may suffer a more aggressive prognosis of T2DM and require more aggressive treatment. Studies show that treatment with intensive insulin therapy for 2.5 weeks can modify the natural history of diabetes. Further, patients with higher body-mass index had higher rates of remission using this therapy [37]. Understanding the disease progression in high-risk populations and further screening of these populations for certain T2DM biomarkers may equip scientists with the tools to prevent and treat the illness more efficiently and reduce associated costs.

Treating patients with complications resulting from diagnosed diabetes has risen from $174 to $245 billion from 2007 to 2013. This 41% increase in five years illustrates the need to reduce diabetes prevalence in the US. High-risk groups should be targeted to help prevent the occurrence of T2DM and to help diabetics make more informed decisions. Patients are often not aware of the severity of T2DM and methods of prevention. Research shows diabetics often lack the necessary information to make educated decisions about their health 24 and that T2DM is associated with many other diseases such as colorectal and breast cancer [38,39]. Direct interventions or communication-based interventions, are necessary to reduce physical and financial burdens cast by this disease [31]. The most significant behavioral change to reduce T2DM complications is physical activity to control blood sugar levels. With proper care and screening, populations can alter their genetic predisposition through lifestyle behavioral modifications. For example, a reduction of weight can drastically reduce the incidence and complications of T2DM in individuals with a family history of T2DM. Those who are at a higher susceptibility or currently afflicted should be encouraged to communicate with their healthcare provider about increased responsibilities, including doctor visits, physical activity, nutrition management, reading labels, and medication use.

There are multiple stages (primordial, primary, and secondary) of prevention and management for T2DM. Primordial prevention includes increasing public awareness about T2DM and reducing risk factors that can lead to disease in children, youth, and young adults. Primary prevention aims at reducing cases of pre-diabetes that advance to T2DM. Secondary prevention focuses on optimizing diabetes management to prevent further complications but may not be easy to achieve. The 2008 Physical Activity Guidelines for Americans recommends moderate levels of activity to lower the risk of developing T2DM by 30–40% [40]. Coupled with guidelines for adherence to nutritional modifications and better circadian rhythms and sleep, Americans who are pre-diabetic or have the susceptible gene loci would have a high probability of never contracting the disease [24]. However, it is important to consider high-risk patients who may not be able to change their lifestyles and might require additional medication [12] or considering the benefits to costs ratio of implementing more rigorous screening and management practices.

Scientists hope that they will be able to isolate SNPs to predict genetic susceptibility for type 2 diabetes. SNPs have been used by grouping individuals based on the number of risk alleles and calculating the area under the receiver operating characteristic curve (AUC). A meta-analysis of more than twenty studies shows limited use of SNPs in predicting individual’s risk of disease. These genetic prediction models could be improved with increased precision of the diagnosis of T2DM and by increasing the SNPs collected (non-European populations) [30].

Focusing on patients who are at a higher risk for T2DM, while still promoting population-based prevention methods, can reduce the socioeconomic burden of diabetes and the prevalence of the disease. Screening for genetic risk factors of T2DM should be considered when implementing prevention methods, especially in high-risk populations. Promoting interventions through communication may further reduce diabetes complications. Future studies need to examine high-risk populations genetic and environmental predisposing risk factors in the etiology of T2DM to reduce the overall burden of the disease in the US. Although there are no direct estimates of the benefits of screening and other prevention techniques, help in early detection and prove beneficial in determining the further progress of the disease. On a wider scale, it can prove to be more cost-effective compared to other post-complication treatments. However, we need more direct studies determining these effect and benefits in further details.

## Abbreviations

T2DM: Type 2 Diabetes Mellitus
USPSTF: U.S. Preventive Services Task Force
MODY: Maturity-Onset Diabetes of the Young
MZ: Monozygotic Twins
ADA: American Diabetes Association
IDF: International Diabetes Federation
FPG: Fasting Plasma Glucose
OGTT: Oral Glucose Tolerance Test
A1C: Haemoglobin A1C
SNPs: Isolate Single Nucleotide Polymorphisms
AUC: Area Under the Receiver Operating Characteristic Curve
BPA: Bisphenol A
